# Modulation of Alveolar Macrophage Activity by Eugenol Attenuates Cigarette-Smoke-Induced Acute Lung Injury in Mice

**DOI:** 10.3390/antiox12061258

**Published:** 2023-06-11

**Authors:** Maria Clara Barbosa-de-Oliveira, Paolo Oliveira-Melo, Marcos Henrique Gonçalves da Silva, Flávio Santos da Silva, Felipe Andrade Carvalho da Silva, Bruno Vinicios Silva de Araujo, Moacir Franco de Oliveira, Aristides Tadeu Correia, Sidnei Miyoshi Sakamoto, Samuel Santos Valença, Manuella Lanzetti, Martina Schmidt, Emanuel Kennedy-Feitosa

**Affiliations:** 1 Morphophysiopharmacology Laboratory, Department of Health Sciences, Federal University of the Semi-Arid Region, Mossoró 59625-900, Brazil; maria.oliveira49268@alunos.ufersa.edu.br (M.C.B.-d.-O.); flavio.santos@ufersa.edu.br (F.S.d.S.); felipe.silva62379@alunos.ufersa.edu.br (F.A.C.d.S.); bruno.araujo73880@alunos.ufersa.edu.br (B.V.S.d.A.); sakamoto@ufersa.edu.br (S.M.S.); 2 Laboratório de Pesquisa em Cirurgia Torácica, Faculdade de Medicina HCFMUSP, Universidade de Sao Paulo, Sao Paulo 05508-220, Brazil; paololiveiramelo@usp.br (P.O.-M.); aristides.correia@incor.usp.br (A.T.C.); 3 Department of Clinical Medicine, Federal Fluminense University (UFF), Niterói 24000-000, Brazil; marcos_goncalves@id.uff.br; 4 Department of Animal Science, Federal University of the Semi-Arid Region, Mossoró 59625-900, Brazil; moacir@ufersa.edu.br; 5 Institute of Biomedical Sciences, Federal University of Rio de Janeiro (UFRJ), Rio de Janeiro 21941-590, Brazil; samuelv@icb.ufrj.br (S.S.V.); manuellalanzetti@icb.ufrj.br (M.L.); 6 Department of Molecular Pharmacology, University of Groningen, Antonius Deusinglaan 1, Building 3211, Room 406, 9713 AV Groningen, The Netherlands; m.schmidt@rug.nl; 7 Groningen Research Institute of Asthma and COPD, GRIAC, University Medical Center Groningen, University of Groningen, 9713 AV Groningen, The Netherlands

**Keywords:** cigarette, macrophages, pulmonary inflammation, oxidative stress, eugenol

## Abstract

This study investigates the role of eugenol (EUG) on CS-induced acute lung injury (ALI) and how this compound is able to modulate macrophage activity. C57BL/6 mice were exposed to 12 cigarettes/day/5days and treated 15 min/day/5days with EUG. Rat alveolar macrophages (RAMs) were exposed to CSE (5%) and treated with EUG. In vivo, EUG reduced morphological changes inflammatory cells, oxidative stress markers, while, in vitro, it induced balance in the oxidative stress and reduced the pro-inflammatory cytokine release while increasing the anti-inflammatory one. These results suggest that eugenol reduced CS-induced ALI and acted as a modulator of macrophage activity.

## 1. Introduction

Acute lung injury (ALI) is a recurrent clinical disorder in critically ill patients. Characterized by inflammation and non-cardiogenic pulmonary edema, ALI directly impacts the morbidity and mortality of patients with severe acute respiratory failure [1].

Cigarette smoke contains oxidants/free radicals [2] and, in the airways, it directly recruits and activates inflammatory cells such as neutrophils, lymphocytes, and macrophages [3,4]. Once recruited, these cells will provide cellular mechanisms that result in the endogenous release of reactive oxygen species (ROS), that links smoking with inflammation and oxidative stress [3]. In addition, smoking promotes pathophysiological changes characteristic of ALI, including increased permeability of the pulmonary epithelial, endothelial injury, and alteration of platelet function [5,6].

Acute lung injury is associated with endothelial and epithelial dysfunction [7,8], resulting in the migration of various components such as protein, cells, and cellular debris in the tissue. The loss of the alveolar–capillary membrane integrity makes the cellular migration of neutrophils and macrophages easier, for example, and increases the proinflammatory/cytotoxic proteins [9]. Macrophages occur in almost all tissues in the body [10]. In the lungs, alveolar macrophages play a key role in both acute and chronic lung injury. Those cells are able to generate reactive oxidants species, such as reactive oxygen species (ROS) and reactive nitrogen species (RNS), which are over-released during chronic inflammation, leading to oxidative stress [5]. Macrophages especially are increased by up to 10 times in the airway, and lung parenchyma in chronic obstructive pulmonary disease (COPD) patients [6]. Although acute exposure to cigarette smoke does not cause emphysema, a chronic and graver condition of COPD, the morphological and functional alterations of the lung parenchyma are observed from acute exposure to cigarette smoke, where inflammatory and redox markers are present since the onset of the pulmonary insult [11].

These cells have functional and phenotypic plasticity, being able to polarize into M1 macrophages, which express pro-inflammatory mediators, such as tumor necrosis factor alpha (TNF-α) and interleukin 8 (IL-8), or into M2 macrophages, which release anti-inflammatory cytokines, such as interleukin 10 (IL-10) and transforming growth factor β (TGF-β) [4,12]. In general, macrophages can release ROS and RNS during inflammation, leading to oxidative stress [13].

Bioactive compounds derived from several species of plant origin have demonstrated antioxidant and anti-inflammatory properties in acute lung injury and may contribute to the prevention of inflammatory diseases [14]. In this context, eugenol (4-allyl-2-methoxyphenol), a phenylpropanoid found in cloves (Syzygium aromaticum), cinnamon (Cinnamomum verum), and coffee, among other foods [15], has already demonstrated anti-inflammatory effects on LPS-induced lung injury in rats [16] and in vitro antioxidant properties [17,18,19]. However, there are no investigations demonstrating the anti-inflammatory and antioxidant effects of eugenol (EUG) on cigarette-smoke-induced acute lung injury, nor its immunomodulatory activity on macrophages.

A demonstration of the pharmacological potential of eugenol as an antioxidant and anti-inflammatory agent raises discussions about new possibilities in the treatment of smoking-related respiratory diseases. In this sense, the present study shows the anti-inflammatory and antioxidant effects of eugenol in acute lung injury caused by cigarette smoke inhalation, and its role in the modulation of alveolar macrophage activity.

## 2. Materials and Methods

### 2.1. Reagents

All reagents were purchased from Sigma Chemical (Sigma Chemical Co., St Louis, MO, USA) and maintained under optimum conditions. Eugenol purity of 99% was confirmed by manufacturer (Sigma Chemical Co., St Louis, MO, USA).

### 2.2. Animals

6 Wistar rats (200–250 g) were used in the in vitro study and 35 male C57BL/6 mice (18–22 g) were used in the in vivo study. All animals were fed with chow and had free access to water in a controlled environment (18–22 °C, 50 to 70% relative humidity, and 12/12 h light/dark cycle). All methods and protocols involving animal use used in this study were approved by the Ethics Committee on Animal Use (CEUA/UFERSA) (23091.007638/2018-95).

### 2.3. Experimental Design

#### In Vivo Cigarette Smoke Exposure and Treatment

C57BL/6 mice were assigned to four groups (n = 8–9/group): Control, Acute Lung Injury (ALI), Acute Lung Injury + Eugenol (ALI + EUG), and Eugenol (EUG). ALI and ALI + EUG groups were exposed to 12 commercial cigarettes (10 mg of tar, 0.9 mg of nicotine, and 10 mg of carbon monoxide) per day for 5 days, through an inhalation chamber as previously described [20]. Control and EUG groups were exposed to ambient smoke-free air. At the end of each day, mice were treated with 100 mg/mL EUG (ALI + EUG and EUG groups) or with vehicle (Control) by inhalation. EUG treatment was administered in aerosol form for 15 min once per day in a designated chamber (21 cm wide, 20 cm long, and 30 cm high) attached to a nebulizer (Ultrasonic Nebulizer RespiraMax® NS x® Omron Healthcare, Jundiai, Brazil) [21].

### 2.4. In Vivo Experimental Protocols

#### 2.4.1. Tissue Processing and Histological Analysis

Twenty-four hours after the end of the fifth day of the experimental procedure, the animals were anesthetized via inhalation with sevoflurane and euthanized by cervical dislocation. Bronchoalveolar lavage (BAL) fluid was collected by washing the pulmonary airspaces with buffered saline solution (3 × 0.5 mL) in order to determine the total number of leukocytes using a Neubauer chamber [22].

The mice’s right ventricles were perfused with saline to remove lung’s blood. Left lungs were collected and transferred to a buffered formalin-fixing solution (10%; pH 7.2) before embedding in paraffin for histological processing and slide preparation. Then, 5 µm lung sections were performed and stained with hematoxylin and eosin (H&E).

#### 2.4.2. Morphological Analysis

The histopathological analysis was performed using the adaptation of inflammation score previously described [23]. From each lung slice, 10 images (40×) of random and non-coincidental fields of the lung parenchyma were captured and were analyzed by three different blinded investigators. The lung parenchyma images were scored according to the following lung inflammation score criteria: 0, normal tissue; 1, minimal inflammatory change (presence of inflammatory cells without architecture changes); 2, no obvious/minimum damage to lung architecture; 3, alveolar septa thickening; 4, formation of nodules or areas of pneumonitis that distort normal architecture; and 5, total field obliteration [23].

Airway perimeter was assessed by determining the bronchoconstriction index (BCI). For this purpose, images of 7–10 different bronchi were captured at 400x magnification on each slide stained with H&E. BCI was quantified using the number of intercepts that cross the basement membrane (NI) and the number of points that reach the airway lumen (L) by the M42 reticulum. BCI was calculated by the following equation: IBC = NI/√L [24]. Only bronchi whose ratio between the largest and smallest diameter of the ellipse that described its lumen did not exceed 1.25 were accepted.

The measurement of the bronchial epithelium height (µm) was performed from the basement membrane to the end of the cell, in at least 4 different points of the same bronchus, using the ImageJ 1.46r software.

Alveolar macrophage and neutrophil numbers were estimated by counting ten random, non-coincidental fields of lung sections using a 100× objective lens. Two investigators performed morphometry by counting coded sections.

### 2.5. In Vitro Experimental Procedures

#### 2.5.1. Preparation of Cigarette Smoke Extract (CSE)

Smoke from 2 commercial cigarettes (10 mg of tar, 0.9 mg of nicotine, and 10 mg of monoxide) was bubbled into 10 mL of DMEM with 1% FBS with an apparatus fitted with a syringe (1 cigarette/2 min). The resulting solution, defined as 100% CSE, was diluted in DMEM to obtain CSE concentrations of 1; 2.5; 5; and 10% [25].

#### 2.5.2. In Vitro Exposure to Cigarette Smoke Extract (CSE) and Treatment

For this step, rat alveolar macrophages (RAMs) were obtained from Wistar rats. Bronchoalveolar lavage (BAL) fluid was collected by washing the lungs with saline solution (0.9%) (10 × 5 mL). BAL was centrifuged (600 rpm, 10 min, 4 °C) and the supernatant discarded. The cell pellet was resuspended in standard culture medium (DMEM with 1% FBS), plated (1 × 10^6^ cells/well) in triplicate in a final volume of 1 mL of medium, and maintained under controlled conditions (37 °C, 5% CO_2_) for 1 h in order for macrophages to adhere to the bottom of the well. After this interval, the medium was collected, the well was washed with PBS, and the RAM were exposed to different concentrations of CSE (1; 2.5; 5; or 10%) and remained for 1 h under controlled conditions (37 °C, 5% CO_2_). After 1 h, the supernatant was collected for the toxicity test.

After choosing the most viable concentration based on the toxicity test, the same procedure was performed, and the RAM were plated again under the same conditions and steps described above. The standard culture medium was replaced by medium containing 5% CSE in the presence or absence of EUG (10, 30, or 100 μg/mL). In control condition, RAM were exposed only to DMEM. This experiment was performed three times. At the end of the treatment, the supernatant was collected for biochemical and immunological analysis.

### 2.6. Analysis

#### 2.6.1. Cytotoxicity Assay (MTT)

Cellular toxicity was measured using the MTT ([3(4,5-dimethyl-thiazoyl2-yl])2,5 diphenyl-tetrazolium bromide) assay. After exposure to CSE concentrations (1, 2.5, 5, and 10%), alveolar macrophages were incubated with MTT at 37 °C for 4 h. Then, the medium was removed and the formazan crystal was solubilized in dimethylsulfoxide (DMSO). The assay was read at 570 nm.

#### 2.6.2. Redox Imbalance Markers

Lung homogenate (ex vivo) and plate supernatant (in vitro) were used for biochemical assays. Superoxide dismutase (SOD) activity was assayed by monitoring the inhibition of adrenaline auto-oxidation at a wavelength of 480 nm [26]. Catalase activity (CAT) was performed by monitoring the rate of decrease of hydrogen peroxide concentrations at a wavelength of 240 nm [27]. Levels of ROS were assessed by reacting blue nitrotetrazolium salt (NBT) at 630 nm [28]. Malondialdehyde (MDA) concentration was measured by the thiobarbituric acid reactive substance assay (TBARS/EMD Millipore, Billerica, MA, USA) at 532 nm [16].

#### 2.6.3. Inflammatory Markers

Interleukin-10 (IL-10) and keratinocyte chemoattractant (KC) levels were quantified using a specific enzyme immunoassay (ELISA) according to the manufacturer’s instructions (PreproTech Inc., Rocky Hill, NJ, USA) and the standard protocol. Myeloperoxidase (MPO) activity in BAL was measured using hydrogen peroxide, hexadecyltrimethylammonium bromide (HTAB; Cat. No. H5882, Sigma-Aldrich, St Louis, MO, USA), and tetramethylbenzidine (TMB; Cat. No. 860336, Sigma-Aldrich, St Louis, MO, USA) with a wavelength of 630 nm [29].

### 2.7. Statistical Analysis

The statistical analysis was performed using the GraphPad Prism 7 software (San Diego, CA, USA). All the data are expressed as means ± S.E.M. (standard error of the mean). Analysis of variance (one-way ANOVA) was used, followed by the Holm–Sidak or Student–Newman–Keuls method of multiple comparisons, when appropriate. The results considered statistically significant had a probability of null hypothesis lower than 5% (*p* < 0.05).

## 3. Results

### 3.1. Morphology

**Figure 1:**
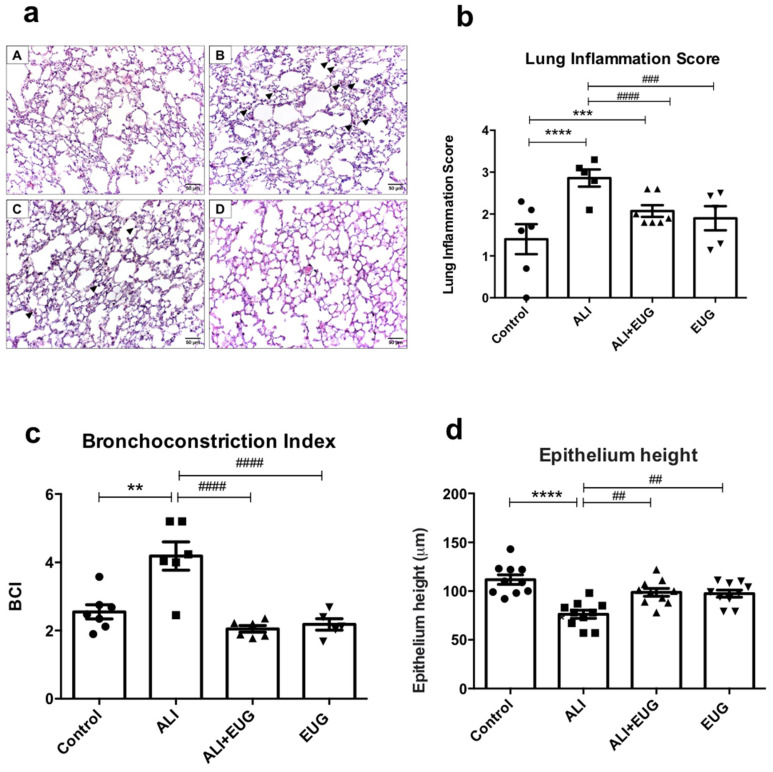
Morphological analysis. (**a**) Photomicrographs of lung sections stained with hematoxylin and eosin: (**A**) control group; (**B**) mice exposed to 12 cigarettes per day for 5 days (ALI); (**C**) mice exposed to 12 cigarettes per day (5 d) and treated with eugenol 100 mg/mL for 5 days (ALI + EUG); and (**D**) EUG group (mice treated with eugenol 100 mg/mL only). Arrowheads, represent leukocytes in alveoli or parenchyma. (**b**) Lung injury scores of the groups (arbitrary values). (**c**) Bronchoconstriction Index (BCI). (**d**) Bronchial epithelium height. ** *p* < 0.01, *** *p* < 0.001, and **** *p* < 0.0001 when compared to the control group. ^##^ *p* < 0.01, ^###^ *p* < 0.001, and ^####^ *p* < 0.0001 when compared to ALI. Values for all measurements are expressed as means ± SEM. *p* < 0.05 was considered statistically significant.

In the histological analysis, the control and EUG groups presented a preserved lung parenchyma with thin alveolar septa and the rare presence of leukocytes. The ALI group showed leukocyte infiltration (arrowheads) in the alveoli and in the parenchyma. ALI + EUG showed a lung parenchyma similar to the control group, with less leukocyte infiltration (arrowheads) (Figure 1a). Furthermore, the lung inflammation score in the ALI group (2.86 ± 0.08) was higher compared to the control group (1.39 ± 0.18) (*p* < 0.0001). ALI + EUG (2.07 ± 0.07) showed a lower lung inflammation score than the ALI group (*p* < 0.0001) (Figure 1b).

When evaluating the bronchoconstriction index (BCI), we observed that the BCI was higher in the ALI group (4.18 ± 0.41) compared to the control (2.55 ± 0.20). On the other hand, the ALI + EUG (2.01 ± 0.12) and EUG (2.18 ± 0.23) groups had lower BCI in relation to ALI (Figure 1c). The ALI group (76.30 ± 4.15) had a lower epithelium height (µm) compared to the control group (111.8 ± 4.94), and the ALI + EUG group (98.80 ± 3.99) recovered the epithelium height in relation to the ALI group (Figure 1d).

### 3.2. Ex Vivo Inflammatory Markers

**Figure 2:**
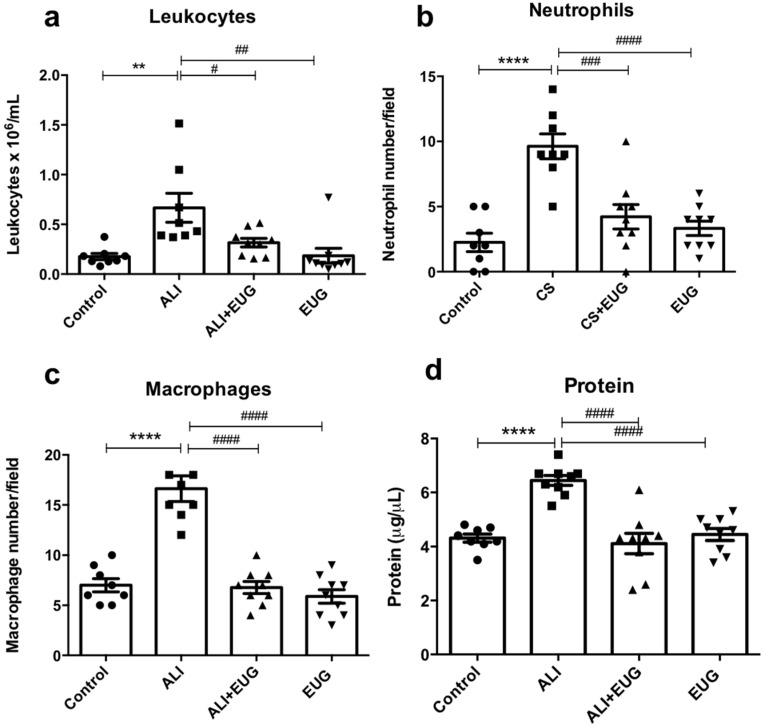
Effects of EUG on inflammatory markers in vivo. The total number of (**a**) leukocytes (10^6^/mL), (**b**) neutrophils, (**c**) macrophages per field quantified by morphometry, and (**d**) concentration of protein in BALF. ** *p* < 0.01 and **** *p* < 0.0001 when compared to the control group. # *p* < 0.05, ## *p* < 0.01, ### *p* < 0.001 and #### *p* < 0.0001 when compared to the ALI group. Values for all measurements are expressed as means ± SEM. *p* < 0.05 was considered statistically significant.

Exposure to cigarette smoke increased BAL leukocyte infiltrate two-fold compared to the control group (total leukocytes: ALI—0.66 ± 0.14 vs. 0.17 ± 0.03 106 cells/mL). EUG reduced the migration of total leukocytes (0.31 ± 0.04 vs. ALI: 0.66 ± 0.14 cells/field), neutrophils (4.22 ± 0.93 vs. ALI: 9.62 ± 0.96 cells/field), and macrophages (6.77 ± 0.59 vs. ALI: 16.63 ± 1.28 106 cells/field) in 47%, 44%, and 41%, respectively (Figure 2a–c). The concentration of protein in the BALF was significantly increased in the ALI group when compared to the control group (6.44 ± 0.18 vs. 4.31 ± 0.14 ug/uL). EUG reduced the concentration of protein in the BALF (4.11 ± 0.37 ug/uL) (Figure 2d).

### 3.3. Ex Vivo Redox Markers

**Figure 3:**
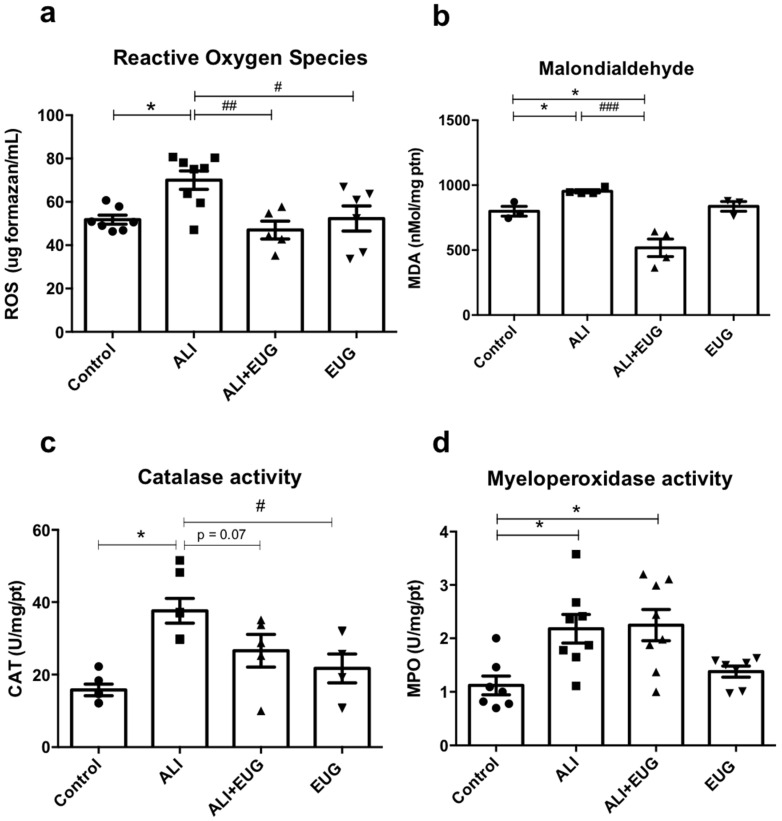
Effects of EUG on ex vivo redox markers. (**a**) Levels of reactive oxygen species (ROS) measured by the NBT assay. (**b**) The equivalent concentration of malondialdehyde (MDA) measured by the TBARS assay. (**c**) Catalase activity (CAT) measured by H_2_O_2_ consumption. (**d**) Myeloperoxidase (MPO) activity measured by the hexadecyltrimethylammonium bromide method. * *p* < 0.05 when compared to the control group. ^#^ *p* < 0.05, ^##^ *p* < 0.01, and ^###^ *p* < 0.001 when compared to the LPA group. Values for all measurements are expressed as means ± SEM. *p* < 0.05 was considered statistically significant.

Elevated ROS levels were observed in the ALI group (70.0 ± 4.2) in relation to the control group (51.7 ± 2.1; *p* < 0.01), while the ALI + EUG group showed reduced levels of ROS (47.02 ± 4.1; *p* < 0.001) when compared to ALI (Figure 3a). The increase in ROS levels was accompanied by an increase in MDA levels in the ALI group (943.6 ± 18.2) when compared to the control (799.7 ± 37.3; *p* < 0.05). MDA levels were lower in the group treated with EUG (518.0 ± 67.8) when compared to the control group (799.7 ± 37.3) (*p* < 0.001) (Figure 3b). CAT activity increased in the ALI group (34.8 ± 3.2) in relation to the control group (18.9 ± 2.3) (*p* < 0.01), but there was no statistical difference between the ALI and ALI + EUG groups (28.2 ± 4.03) (Figure 3c). When MPO levels were evaluated, the treatment was not able to reduce the increase in this marker of neutrophil infiltration in the ALI + EUG group (2.24 ± 0.2) in relation to the ALI group (2.17 ± 0.2) (Figure 3d).

### 3.4. Cell Viability of RAM

**Figure 4:**
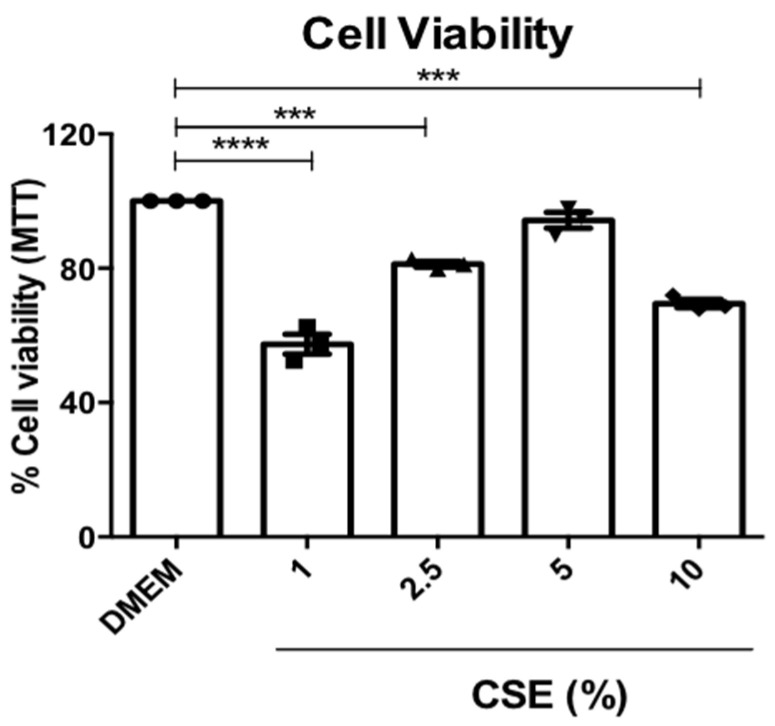
Cell viability of RAM using the MTT method ([3(4,5-dimethyl-thiazoyl2-yl])2,5 diphenyl-tetrazolium bromide). *** *p* < 0.001 and **** *p* < 0.0001 when compared to the DMEM condition. Values for all measurements are expressed as means ± SEM. *p* < 0.05 was considered statistically significant.

Cells exposed to CSE 1, 2, and 10% showed lower cell viability in the toxicity test compared to the standard culture medium. However, in the CSE 5% medium, the MTT test presented similar results to the DMEM, and was chosen to carry out the other in vitro experiments (Figure 4).

### 3.5. In Vitro Inflammatory Markers

**Figure 5:**
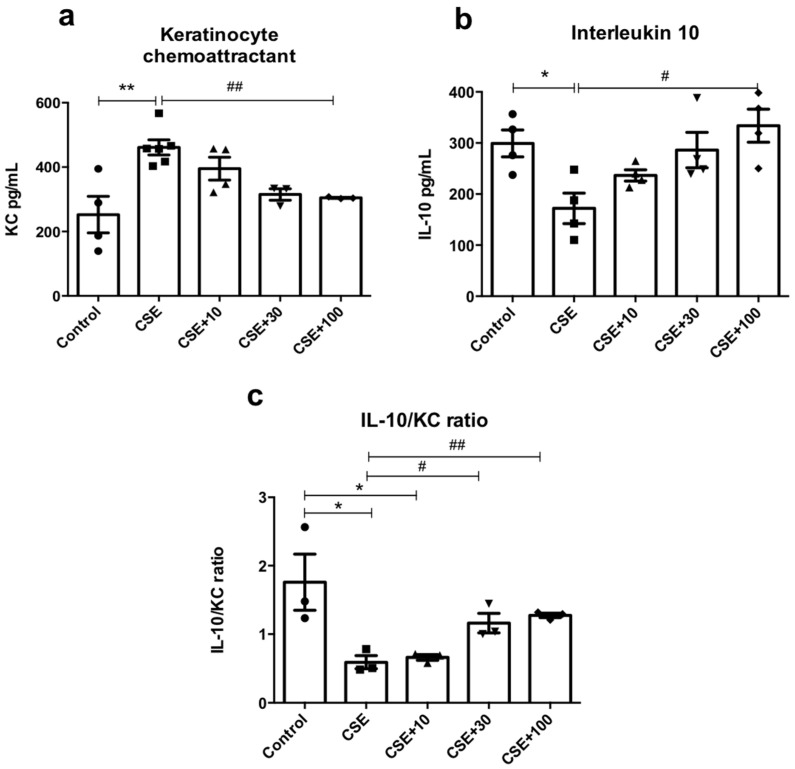
Effects of EUG on inflammatory markers in vitro. (**a**) KC levels; (**b**) IL-10 levels; (**c**) IL-10/KC ratio. * *p* < 0.05 and ** *p* < 0.01 in relation to the control group; ^#^ *p* < 0.05 and ^##^ *p* < 0.01 in relation to the EFC group. Values for all measurements are expressed as means ± SEM. *p* < 0.05 was considered statistically significant.

KC levels increased by 82% in the CSE condition (461.4 ± 23.5; *p* < 0.01) when compared to control (252.4 ± 56.8). EUG (100 μg/mL) reduced KC levels by 34% (304.6 ± 1.70; *p* < 0.01) (Figure 5a). On the other hand, IL-10 levels were reduced by 50% in the CSE condition (CSE: 146.8 ± 22.4 vs. Control: 290.0 ± 35.0; *p* < 0.05). Treatment with EUG (100 μg/mL) recovered IL-10 levels by 127% (333.8 ± 32.2; *p* < 0.05) (Figure 5b). Finally, when the IL-10/KC ratio was evaluated, the CSE condition presented a lower relation in relation to the control (CSE: 0.59 ± 0.09 vs. Control: 1.75 ± 0.40; *p* < 0.05), increasing with treatment (CSE + 30: 1.161 ± 0.14; (*p* < 0.05) and CSE + 100: 1.274 ± 0.03; (*p* < 0.01)) (Figure 5c).

### 3.6. In Vitro Redox Markers

**Figure 6:**
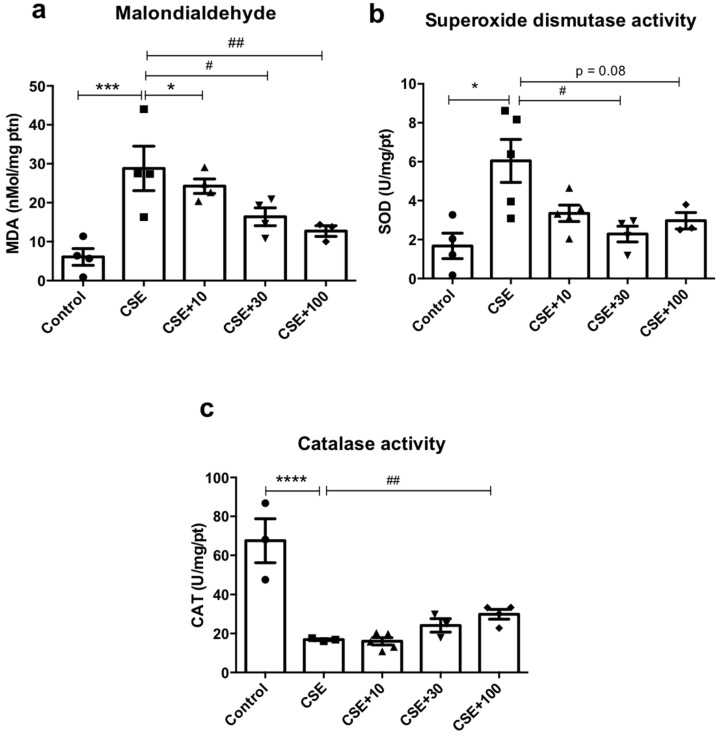
Effects of EUG on in vitro redox markers. (**a**) The equivalent concentration of malondialdehyde (MDA) measured by the TBARS assay. (**b**) Superoxide dismutase (SOD) activity measured by monitoring the inhibition of adrenaline autooxidation. (**c**) Catalase activity (CAT) measured by H_2_O_2_ consumption. * *p* < 0.05, *** *p* < 0.001, and **** *p* < 0.0001 when compared to the control group. ^#^ *p* < 0.05 and ^##^ *p* < 0.01 when compared to the CSE group. Values for all measurements are expressed as means ± SEM. *p* < 0.05 was considered statistically significant.

MDA levels in the CSE condition increased by five times (32.9 ± 5.51; *p* < 0.01) in relation to the control (6.10 ± 2.14). In the presence of EUG, only cells treated with 100 μg/mL showed reduced levels of MDA (12.7 ± 1.37; *p* < 0.05) (Figure 6a). SOD activity was higher in the CSE condition than in the control condition (6.04 ± 1.10 vs. 1.67 ± 0.65; *p* < 0.05), being significantly reduced with the treatment of EUG 30 μg/mL (2.28 ± 0.40, *p* < 0.5) (Figure 6b). In addition, CAT activity decreased in the CSE (16.8 ± 0.46) compared to control (67.5 ± 11.3) (*p* < 0.0001) and increased in the CSE + EUG 100 μg condition (29.9 ± 2.48) in relation to the CSE condition (*p* < 0.01) (Figure 6c).

## 4. Discussion

This is the first study to demonstrate the role of eugenol in modulating alveolar macrophages and its antioxidant and anti-inflammatory effects in a model of lung injury induced by cigarette smoke inhalation.

Acute inhalation of cigarette smoke induces inflammatory changes in the lungs of mice, manifested by a thickening of the alveolar septa, areas of inflammatory infiltrate, and alveolar hemorrhage. Eugenol treatment improved these inflammatory histopathological changes and significantly decreased the mean lung inflammation score, which represents a reduction in lung injury due to eugenol’s anti-inflammatory properties. This finding corroborates other studies which show an anti-inflammatory activity of eugenol in LPS-induced lung inflammation models [10] and in human macrophages, reducing the release of IL-1β, TNF-α, and cyclooxygenase (COX) [30].

It is well-documented that acute exposure to cigarette smoke can cause acute bronchoconstriction [30,31]. As previously demonstrated, BCI is a morphometric parameter for the indirect assessment of bronchoconstriction [32]. Eugenol treatment reduced BCI, suggesting a potential bronchodilator effect of this constituent. Furthermore, this finding is supported by the bronchodilator property of eugenol in asthma models described in the literature, in which this compound was able to reverse contractions in the airways induced by bronchoconstrictor pharmacological agents [33].

In acute lung injury, there is a disruption of the pulmonary endothelial and epithelial barriers. The latter constitutes the initial barrier against modulating agents of airway smooth muscle (ASM) tone [34]. In this sense, it has been previously demonstrated that cigarette smoke reduces the integrity of the epithelial layer [35], damaging this structure, and, consequently, exposing spasmogenic agents to ASM. This exposure, associated with the reduction in relaxation induced by epithelial-derived relaxing factors, results in increased airway responsiveness [13]. Thus, the reduction of the epithelium height in the ALI group may explain, in part, the increase in the bronchoconstriction index induced by acute exposure to cigarette smoke. Treatment with eugenol protected the bronchial epithelium height, thus demonstrating a reduction in epithelial damage induced by CS exposure, which may explain the reduction in BCI by eugenol.

Leukocytes produce and release oxidants in response to inflammatory stimuli. These inflammatory cells, particularly macrophages and neutrophils, are recruited and activated during lung inflammation induced by cigarette smoke inhalation [3,4,36]. In the present study, exposure to cigarette smoke increased leukocyte migration in bronchoalveolar lavage and lung tissue. Eugenol treatment modified the cellular profile in BAL/lung parenchyma, reducing the total number of leukocytes, mainly macrophages and neutrophils. Since these cells produce and release oxidants in response to the inflammatory stimulus and contribute to maintain the state of oxidative stress, these results suggest a correlation between the improvement of the redox profile with the ability of eugenol to reduce the influx of leukocytes in BAL fluid, as already demonstrated by Butler et al. (2018), who showed a correlation between the number of macrophages in BAL and lung tissues’ damage [37].

Neutrophils have an important role in pulmonary inflammation and fibrosis by releasing inflammatory mediators such as MPO [38], whose activity, in our study, was increased in the ALI group. Interestingly, the reduction in the neutrophil influx was not accompanied by a decrease in MPO activity. Although counterintuitive, this has been reported in another study of LPS-induced ALI [39]. This finding leads us to believe that the anti-inflammatory and antioxidant effect of eugenol in our model may not be mediated by neutrophils, but by the modulation of macrophages, since these are cells that act in the first line of defense against inhaled particles [40].

Furthermore, it has already been described that oxidative stress can have direct and indirect effects on macrophage function and that these cells are able to response to several toxicant-induced injuries through pro-inflammatory mediators such as ROS, RNS, IL-6, IL-18, TNF-a, and proteases, and all of them will induce and/or amplify the inflammatory response [9,41].

According to Lee et al. [30], smoking has been shown to affect the function and phenotype of macrophages, that consist of two states of polarization. M1 macrophages (classically activated) express high levels of pro-inflammatory cytokines, such as tumor necrosis factor (TNF)-α, IL-8, and IL-6. In contrast, the M2 phenotype (alternatively activated macrophages) expresses CD206 and anti-inflammatory cytokines, including IL-10 and TGF-β [4,13]. M2 cells antagonize the effects of M1 cells and promote tissue repair after inflammatory injury, acting as regulators of the inflammatory process [42].

During macrophage activation, the production of IL-10 depends on the polarization state, being more produced by M2 macrophages [10]. Furthermore, IL-10 is required for macrophage polarization to the M2 phenotype, as it makes macrophages more sensitive to being targeted in this polarization state [43]. In this context, IL-10 is one of the markers of M2a cells—a subtype of M2 macrophages—in humans and mice [42]. However, an important association was shown between serum nicotine levels and IgE sensitization with potential dose-dependent relationships [44]. It can suggest that M2 polarization is able to lead to the induction of IgE and an inflammatory response in high exposures to cigarette smoke, resulting in M2 response suppression [45].

Several signaling pathways have been implicated in the polarization of macrophages mediated by cigarette smoke. To address this, we isolated alveolar macrophages from rats instead of mice. Although it may constitute a potential limitation of our study (since we opted for another species in relation to the in vivo model), RAMs are cells which show some advantages such as higher cell viability and a morphology closer to human alveolar macrophage (vacuolization, size, and lipid content) [46].

Here, we showed that macrophage exposure to CSE resulted in increased KC levels and reduced IL-10 levels, and that treatment with 100 μg/mL EUG had an opposite effect, reducing the levels of KC, a pro-inflammatory cytokine homologous to IL-8 in humans, and increasing the IL-10 levels. This modulation may be responsible for regulating the immune response and preventing damage [37,47]. The increase in IL-10 levels after eugenol treatment contributes to reducing the release of pro-inflammatory cytokines, since IL-10 suppresses the production of these cytokines by macrophages [36]. Therefore, the increase in the IL-10/KC ratio, markers of the M2 and M1 phenotype, respectively, after treatment with eugenol contributes to supporting the hypothesis that the anti-inflammatory and antioxidant activity of eugenol could be via macrophage modulation.

Lung damage is associated with oxidative stress and has been implicated in extracellular matrix remodeling and lipid peroxidation [48], contributing to cigarette-smoke-induced inflammatory changes in the lungs. The reduction of MDA concentrations (ex vivo and in vitro), a cytotoxic product of lipid peroxidation, by eugenol suggests that this compound has an antioxidant effect and, therefore, reduces oxidative damage in lung tissue. This mechanism may also help to explain the observed improvements in lung histoarchitecture.

The redox imbalance resulting from the macrophage response (in vitro) was assessed by the antioxidant enzymatic activity of SOD and CAT and the levels of MDA. Cigarette smoke releases oxidants such as the superoxide anion which is converted by SOD to hydrogen peroxide [26]. This explains the increased activity of SOD by macrophages when these cells were exposed to CSE, since SOD acts in the first line of defense against oxidants and is the main defense factor against oxygen radicals during oxidative stress [38,49]. The attenuation of the redox imbalance by the treatment with 30 μg/mL EUG also explains the reduced need for SOD activity in this response.

SOD action on the superoxide anion results in the formation of hydrogen peroxide (H_2_O_2_), which is added to the H_2_O_2_ released by cigarette smoke itself [48]. In this sense, we suggest that there is an increase in the need for CAT activity after exposure to cigarette smoke, since SOD activity associated with the H_2_O_2_ released from smoke substantially raises the levels of H_2_O_2_ in the lungs.

Eugenol treatment improved the oxidant/antioxidant balance, promoting an increase in CAT activity and, consequently, leading to a greater conversion of H_2_O_2_ into the hydroxyl radical and water, as well as a possible reduction in the release of H_2_O_2_ by alveolar macrophages. This likely reduced lipid peroxidation, resulting in lower levels of MDA [50].

These findings suggest that eugenol has an antioxidant activity via the activation of the enzymatic antioxidant system, acting preferentially via CAT and reducing H_2_O_2_ levels. These results are supported by previous studies that demonstrated the antioxidant effects of eugenol in in vivo and in vitro models [51,52].

## 5. Conclusions

In summary, our study suggests that eugenol has antioxidant and anti-inflammatory activity, being able to attenuate acute lung injury induced by cigarette smoke and reducing morphological changes in the lungs of mice. These effects occur, mediated by the reduction of the redox imbalance in vivo and in vitro, especially by improving enzymatic antioxidant defenses. Furthermore, eugenol especially modulates macrophage activity, resulting in less inflammation and, consequently, less redox imbalance, suggesting, therefore, that the anti-inflammatory and antioxidant effects of eugenol are mediated mainly through the modulation of those cells. Thus, eugenol is a compound with potential pharmacological applications in the treatment of acute lung injury.

## Data Availability

The data that support the findings of this study are available from the corresponding author, Emanuel Kennedy-Feitosa, upon reasonable request.
